# Activation of β2-Adrenoceptor Attenuates Sepsis-Induced Hippocampus-Dependent Cognitive Impairments by Reversing Neuroinflammation and Synaptic Abnormalities

**DOI:** 10.3389/fncel.2019.00293

**Published:** 2019-07-10

**Authors:** Man-Man Zong, Zhi-Qiang Zhou, Mu-Huo Ji, Min Jia, Hui Tang, Jian-Jun Yang

**Affiliations:** ^1^Department of Anesthesiology, Jinling Hospital, School of Medicine, Nanjing University, Nanjing, China; ^2^Department of Anesthesiology, Zhongda Hospital, School of Medicine, Southeast University, Nanjing, China; ^3^Department of Anesthesiology, First Affiliated Hospital of Zhengzhou University, Zhengzhou, China

**Keywords:** β2-AR, sepsis, cognition, synaptic plasticity, neuroinflammation

## Abstract

Sepsis-associated encephalopathy induces cognitive dysfunction via mechanisms that commonly involve neuroinflammation and synaptic plasticity impairment of the hippocampus. The β2-adrenoceptor (β2-AR) is a G-protein coupled receptor that regulates immune response and synaptic plasticity, whereas its dysfunction has been implicated in various neurodegenerative diseases. Thus, we hypothesized abnormal β2-AR signaling is involved in sepsis-induced cognitive impairment. In the present study, C57BL/6 mice were subjected to cecal ligation and puncture (CLP) to mimic the clinical human sepsis-associated encephalopathy. The levels of hippocampal β2-AR, proinflammatory cytokines tumor necrosis factor (TNF-α), interleukin-1β (IL-1β), IL-6, cAMP-response element binding protein (CREB), brain derived neurotrophic factor (BDNF), post-synaptic density protein 95 (PSD95), and NMDA receptor 2 B subtypes (GluN2B) were determined at 6, 12, 24 h and 7 and 16 days after CLP. For the interventional study, mice were treated with β2-AR agonist clenbuterol in two ways: early treatment (immediately following CLP) and delayed treatment (on the 8th day following CLP). Neurobehavioral performances were assessed by open field and fear conditioning tests. Here, we found that hippocampal β2-AR expression was significantly decreased starting from 12 h and persisted until 16 days following CLP. Besides, sepsis mice also exhibited increasing neuroinflammation, down-regulated CREB/BDNF, decreasing PSD95 and GluN2B expression, and displayed hippocampus-dependent cognitive impairments. Notably, early clenbuterol treatment alleviated sepsis-induced cognitive deficits by polarizing microglia toward an anti-inflammatory phenotype, reducing proinflammatory cytokines including IL-1β, TNF-α, and up-regulating CREB/BDNF, PSD95, and GluN2B. Intriguingly, delayed clenbuterol treatment also improved cognitive impairments by normalization of hippocampal CREB/BDNF, PSD95, and GluN2B. In summary, our results support the beneficial effects of both early and delayed clenbuterol treatment, which suggests that activation of β2-AR has a translational value in sepsis-associated organ dysfunction including cognitive impairments.

## Introduction

Sepsis is defined as the host’s reaction to infection and characterized by a systemic inflammatory response with important clinical implications (Singer et al., 2016). Sepsis-associated encephalopathy (SAE) represents diffuse cerebral dysfunction during sepsis, which frequently develops long-term cognitive impairments, including attention, memory, executive function, and speed of information processing (Gofton and Young, 2012), and some skills do not be improved completely in septic patients after 1 or 2 years of follow-up (Comim et al., 2012), which is associated with poor patient outcomes, such as prolonged hospital stay, reduced quality of life, loss of social dependence, and even increased mortality (Zhang et al., 2014; Pan et al., 2019). Unfortunately, there is no specific therapy for sepsis-induced cognitive impairments (Robba et al., 2018), in part because of our limited knowledge of its underlying pathophysiological mechanisms. Thus, deeper understanding of its etiological mechanisms is necessary for seeking novel and effective therapeutic strategies.

Sepsis-associated encephalopathy is a diffuse brain disorder involving multiple brain regions and the hippocampus is one of the most vulnerable parts, which is highly susceptible to ischemia, anoxia, and inflammation (Annane, 2009). The hippocampus plays a key role in Learning and memory processes and this area is a primary site of adult neurogenesis (Zhao et al., 2008). It has been demonstrated that hippocampus damage strongly destroys various kinds of learning and memory (Zheng et al., 2015).

The β2-adrenoceptor (β2-AR) is a G-protein coupled cell membrane receptor, which involves activation of the cyclic adenosine monophosphate/protein kinase A (cAMP/PKA) cascade, resulting in phosphorylating cyclic AMP response element binding protein at Ser-133 (pCREB) and thus facilitating the transcription of key proteins necessary for synaptic plasticity such as brain-derived neurotrophic factor (BDNF) (Karthivashan et al., 2019). The later has a crucial role in memory and cognitive functions (Han R. et al., 2019). In support with this notion, β2-AR activation has been shown to facilitate synaptic plasticity and enhance memory formation (Shi et al., 2018), whereas animal study indicates that genetic deletion of β2-AR results in impaired cognitive modulation by stress or corticosterone (Schutsky et al., 2011). Moreover, another animal study indicates that locally injection of the β2-AR antagonist into the hippocampal CA3 region impaired long-term contextual fear memory and water-maze spatial memory in mice (Zheng et al., 2015). In addition, numberous animal studies that β2-AR has potential antiinflammatory properties and plays an important role in maintaining the immunosuppressive environment within the central nervous system (CNS)(Agac et al., 2018; Xu et al., 2018). Thus, activation of β2-AR has been used for the treatment of many neurodegenerative diseases, such as Alzheimer’s disease (AD) (Wu et al., 2017), Rett syndrome (Mellios et al., 2014), and Parkinson’s disease (Abdelmotilib and West, 2017). However, little is known about the role of hippocampal β2-AR in sepsis-induced cognitive impairments.

Based on these findings, we aimed to investigated whether abnormal β2-AR signaling is involved in sepsis-induced cognitive impairments induced by cecal ligation and perforation (CLP). Furthermore, we hypothesized that the beneficial effects of β2-AR activation is related to its ability to reverse the neuroinflammation and synaptic abnormalities after sepsis.

## Materials and Methods

### Animals

Two hundred and twenty male C57BL/6 mice (3–4 months, 22–25 g) were obtained from the Animal Center of Jinling Hospital, Nanjing, China and efforts were made to minimize the number of mice involved and their suffering as possible as we can. All procedures were carried out based on the Guideline for the Care and Use of Laboratory Animals from the National Institutes of Health (Bethesda, MD, United States). Experiments were performed in accordance with protocols approved by the Ethics Committee of Jinling Hospital, Nanjing University, China. Mice were reared in groups of 3–5 individuals per cage under aseptic conditions maintained at 23 ± 1°C with stable humidity, and had free access to food and water. We begun the experiments after the mice acclimating to the environment for 2 weeks.

### CLP Model

Sepsis was induced by CLP as previously described (Pan et al., 2019). Animals were anesthetized with 2% sodium pentobarbital (50 mg/kg; Sigma Chemical, Co., St. Louis, MO, United States) by intraperitoneal (*i.p.*) injection, followed by a 1-cm midline, under aseptic conditions. Subsequently, the cecum was exteriorized and ligated with a 4.0 silk suture, just below the ileocecal valve. Then, the cecum was gently perforated once with 14-gauge needle and squeezed to extrude little feces through the perforation site. Finally, the cecum was carefully returned to the peritoneal cavity and the laparotomy was closed with 4.0 silk sutures. Mice were subcutaneously resuscitated with normal saline (20 ml/kg) immediately after the surgery. All mice were returned to their cages with free access to food and water. For sham groups, mice were treated identically but without ligation or puncture of the cecum.

### Experimental Design

#### Experiment 1

To determine the time course of hippocampal β2-AR, proinflamamtory mediators and synaptic proteins, the mice were euthanized at following time points: 6, 12, 24 h, 7 days and 16 days after CLP.

#### Experiment 2

Clenbuterol is a brain penetrant β2-AR agonist, not only acts on β2-AR in the brain, but also on other organs such as lung, heart, and kidney. To determine the optimal dose for the treatment of sepsis-induced cognitive impairments, clenbuterol hydrochloride (Sigma, St. Louis, MO, United States) at 0.02, 0.1, or 0.5 mg/kg dissolved in saline solution was injected *i.p.* immediately after operation and once daily for five consecutive days, followed by a 2-days off period, repeated weekly (Dong et al., 2017). Animals in sham groups received the same volume of normal saline at the corresponding time points.

#### Experiment 3

For the early treatment groups, mice received clenbuterol *i.p.* immediately after operation and until the end of the behavioral tests. The experimental design was shown in Figure 4A.

#### Experiment 4

For the delayed treatment groups, mice received clenbuterol *i.p.* on the 8th day after operation and until the end of the behavioral tests. The experimental design was shown in Figure 7A.

### Behavioral and Cognitive Tests

All behavioral procedures were carried out at 2:00–5:00 p.m. in a sound-isolated room. Tests were recorded by the same investigator who was blinded to the grouping of mice as described by our previous studies (Qiu et al., 2016; Mao et al., 2019).

### Open Field Test

The spontaneous locomotor activity of the mice in the open field test was tracked and recorded by a photobeam activity system and software (XR-XZ301, Shanghai Softmaze Information Technology, Co., Ltd., Shanghai, China). The apparatus is made up of a white polyester resin chamber (40 cm × 60 cm × 50 cm high). The mice were placed in the center of the arena and allowed to explore for 5 min, and the total distance moved and the time spent in the center were recorded. The chamber was cleaned with 75% ethanol after each test.

### Fear Conditioning Test

On the day of training, the animals were placed into an enclosed training chamber and allowed to explore for 180 s. Then, the mice were exposed to a tone (30 s, 70 dB, 3 kHz), followed by a 2 s foot shock (0.75 mA). Afterward, the mice were left in the chamber for additional 30 s before being returned to their home cage. Twenty-four hours after the training session, the mice were reexposed in the same chamber for 5 min for the contextual fear conditioning test (a hippocampus-dependent task) (Chen et al., 2010). Two hours later, the mice were placed into a novel chamber which was different in shape, color, and smell from the original chamber for the cued fear conditioning test (a hippocampus-independent task). After a 3 min exploratory period, a training tone (30 s, 70 dB, 3 kHz) was applied for another 3 min and the time of freezing of mice was recorded. The chamber was thoroughly cleaned with 75% ethanol after each trial.

### Immunohistochemistry

The mice were deeply anesthetized with 2% sodium pentobarbital in saline (60 mg/kg, *i.p.*) and perfused transcardially with 0.1M phosphate-buffered saline (PBS, pH 7.4), followed by 4% phosphate-buffered paraformaldehyde (PFA). The brains were removed, post-fixed for 14 h in the same PFA solution, and dehydrated, sequentially embedded in paraffin. Coronal sections of 5 μm thickness were cut for the immunostaining. To quench endogenous peroxidase activity, brain slices were incubated with 3% H_2_O_2_ in methanol for 10 min. After being rinsed with phosphate-buffered saline (PBS), sections were blocked with 10% goat serum for 30 min, and incubated overnight at 4°C with a rabbit antibody against the β2-AR (1:100; Abcam, Cambridge, United Kingdom). After washing with PBS, sections were incubated with biotinylated goat anti-rabbit (1:400; Bioworld Technology, St. Louis Park, MN, United States) for 1 h at room temperature. After washings, the freshly prepared 3,3′-diaminobenzidine (DAB) solution was added and control the reaction time properly. After dyed with hematoxylin, the sections were dipped in 1% hydrochloric acid in alcohol for differentiation. Then, the sections were washed in ammonia and stained blue, rinsed, and coverslipped following gradient dehydration in increasing concentrations of ethanol and cleared with xylene. All the sections were examined under the digital microscope (Motic, Canada) to record the intensity of antigen antibody reaction. The observation of the sections was carried out at magnification of 400X. For immunohistochemistry analysis, three sections per mouse and three mice per group were evaluated for hippocampal region (complete section). Morphometric analysis was carried to observe extent of immunoreactivity. Optical density (OD) was determined as measure of β2-AR expression in hippocampus by analyzing the images with ImageJ 1.51a software (NIH, United States).

### Western Blotting Analysis

The hippocampus was harvested for Western blotting analysis at the indicated time points. The hippocampus was isolated as described previously (Odaira et al., 2019). Each sample was homogenized in the RIPA lysis buffer mixed with 1% protease inhibitor cocktail and 1%PMSF. After centrifuging at 13,000 *g* for 10 min at 4°C, the supernatant was collected and its protein concentration was measured by Bradford assay, 40 μg of proteins per lane were loaded on SDS-PAGE gels and then was transferred to the nitrocellulose blotting membranes. After blocking with 5% non-fat milk in Tris-Buffered Saline with Tween (TBST), membranes were incubated overnight at 4°C in each primary antibody. The primary antibodies used were rabbit anti-β2-AR (1:5000; Abcam, Cambridge, United Kingdom), rabbit anti-CD11b (1:1,000; Abcam, Cambridge, United Kingdom), goat anti-IL-1β (1:500; R&D systems, Minneapolis, MN, United States), rabbit anti-TNF-α (1:1000; Cell Signaling, Danvers, MA, United States), mouse anti-IL-6 (1:200; Santa Cruz Biotechnology, Dallas, TX, United States), mouse anti-CD16 (1:500; Santa Cruz Biotechnology, Dallas, TX, United States), mouse anti-arginase1 (1:500; Santa Cruz Biotechnology, Dallas, TX, United States), mouse anti-CD68 (1:1,000; Abcam, Cambridge, United Kingdom), rabbit anti-CD206 (1:500, proteintech, United States), rabbit anti-pCREB (1:1000; Cell Signaling, Danvers, MA, United States), rabbit anti-CREB (1:1000; Cell Signaling, Danvers, MA, United States), rabbit anti-BDNF (1:500; Santa Cruz Biotechnology, Dallas, TX, United States), anti-pro BDNF (5H8) (1:200; Santa Cruz Biotechnology, Dallas, TX, United States), rabbit anti-postsynaptic density 95 (PSD95) (1:1000; Abcam, Cambridge, United Kingdom), rabbit anti-NMDAR2B (GluN2B) (1:1000; Abcam, Cambridge, United Kingdom), rabbit anti-synaptophysin (1:1000; Abcam, Cambridge, United Kingdom), rabbit anti-β-tubulin (1:1,000; Bioworld, St. Louis Park, MN, United States). After washing in TBST for three times, the membrane were incubated with appropriate secondary antibodies (goat anti-rabbit, goat anti-mouse, or rabbit anti-goat [Bioworld Technology, St. Louis Park, MN, United States]). Chemiluminesence method was used to detect the protein bands and the band intensity were analyzed with Image J Software (Wayne Rasband, National Institutes of Health, United States).

### Statistical Analysis

Statistical analyses were performed by the Statistical Product for Social Sciences (SPSS; version 21.0, IBM). All data are presented as mean ± SEM. Data were tested for normal distribution by the Shapiro–Wilk test. For data that are normally distributed, differences between two groups were compared by independent-samples *t*-tests, whereas differences among multiple means were assessed by one-way, two-way, repeated-measures of analysis of variance (ANOVA) followed by a Bonferroni test as appropriate. The interaction effect of operation (Sham, CLP) and treatment (vehicle, clean) has been indicated in the text as, e.g., the interaction between CLP and clenbuterol treatment and has been analyzed with a two-way ANOVA. Since IL-1β is not normally distributed, Kruskal–Wallis H and Mann–Whitney *U*-tests were used. The survival rate was analyzed by the Kaplan–Meier method and compared by the log-rank test. A *p* < 0.05 was regarded as statistically significant.

## Results

### Time Course of β2-AR Expression Following CLP

**FIGURE 1:**
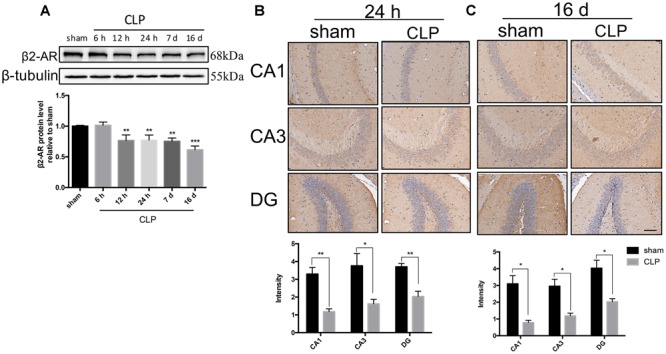
β2-AR in the hippocampus was down-regulated after sepsis. **(A)** Time course of the protein levels of β2-AR in the hippocampus after sepsis. Representative Western blotting bands of β2-AR and quantitative analysis of the relative protein levels. **(B,C)** Representative images and quantification of β2-AR by immunohistochemistry in hippocampus at 24 h and 16 days following CLP. Scale bar = 100 μm. *N* = 3 mice/group. Compared with the sham group, ^∗^*p* < 0.05, ^∗∗^*p* < 0.01, ^∗∗∗^*p* < 0.001. All data are mean ± SEM.

We utilized Western blotting analysis and immunohistochemistry to quantify β2-AR expression in the hippocampus following CLP. Western blotting analysis showed that β2-AR was significantly decreased starting from 12 h and persisted until 16 days following CLP (*F*_5,12_= 17.384, *p* < 0.001; Figure 1A). Consistent with the Western blotting analysis data, the immunohistochemistry confirmed that the expression of β2-AR was significantly decreased at 24 h (CA1: *t* = 5.177, *p* = 0.007; CA3: *t* = 2.918, *p* = 0.043; DG: *t* = 4.691, *p* = 0.009; Figure 1B) and persisted until 16 days following CLP (CA1: *t* = 4.532, *p* = 0.011; CA3: *t* = 3.988, *p* = 0.016; DG: *t* = 3.976, *p* = 0.017; Figure 1C).

### Time Course of Neuroinflammation Markers, CREB/BDNF, and Synaptic Proteins Following CLP

**FIGURE 2:**
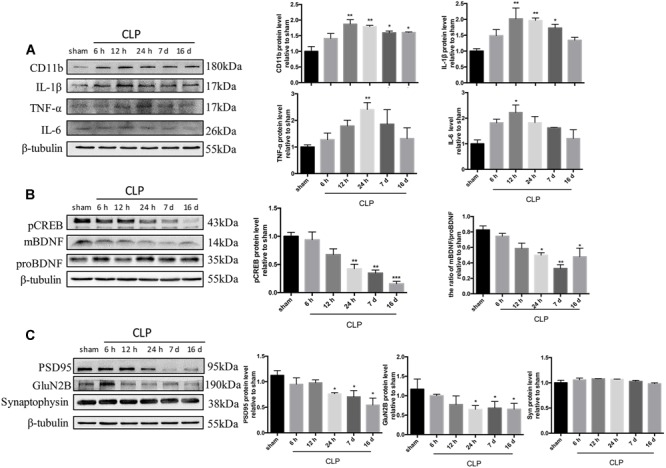
Time course of neuroinflammation markers, CREB/BDNF, and synaptic proteins following CLP. **(A)** Representative Western blotting bands of CD11b, IL-1β, TNF-α, and IL-6 in the hippocampus and quantitative analysis of the relative protein levels. **(B)** Time course of the protein levels of pCREB and mBDNF/proBDNF in the hippocampus after sepsis. Representative Western blotting bands of pCREB and mBDNF/proBDNF and quantitative analysis of the relative protein levels. **(C)** Representative Western blotting bands of PSD95, GluN2B and synaptophysin in the hippocampus and quantitative analysis of the relative protein levels. *N* = 3 mice/group. Compared with the sham group, ^∗^*p* < 0.05, ^∗∗^*p* < 0.01, ^∗∗∗^*p* < 0.001. All data are mean ± SEM.

We next assessed the changes of neuroinflammation markers following CLP. The expression of CD11b, a microglia marker, was significantly upregulated at 12 h, and this upregulation persisted until 16 days after CLP (*F*_5,12_= 8.013, *p* = 0.002; Figure 2A). When compared with sham group, the expressions of inflammatory mediators sush as IL-1β, TNF-α, and IL-6 in the hippocampus were dramatically increased in CLP group: the level of IL-1β rose significantly at 12, 24 h and 7 days following CLP (*F*_5,12_= 4.948, *p* = 0.011; Figure 2A); the IL-6 increased at 12 h following CLP (*F*_5,12_= 3.969, *p* = 0.023; Figure 2A) while the TNF-α increased at 24 h following CLP (*F*_5,12_= 7.330, *p* = 0.002; Figure 2A).

One of the typical signaling pathways downstream of the β2-AR activation involves pCREB at Ser-133 (pCREB) and facilitates the transcription of BDNF. BDNF is synthesized as a precursor molecule (proBDNF), and then cleaved into mature BDNF within endoplasmic reticulum, which plays an important role in synaptic plasticity and neuronal survival (Borodinova and Salozhin, 2017; Li et al., 2019). As shown in Figure 2B, sepsis significantly decreased the expressions of pCREB (*F*_5,12_= 14.95, *p* < 0.001) and the ratio of mBDNF/proBDNF (*F*_5,12_= 8.32, *p* = 0.001) at 24 h and persisted until 16 days following CLP. As shown in Figure 2C, levels of post-synaptic proteins, including PSD95 (*F*_5,12_= 13.08, *p* < 0.001) and GluN2B (*F*_5,12_= 4.601, *p* = 0.014) decreased from 24 h and persisted until 16 days following CLP. However, the level of the presynaptic membrane protein synaptophysin was not altered (*F*_5,12_= 2.038, *p* = 0.145).

### Optimal Dose of Clenbuterol for the Treatment of Sepsis-Induced Cognitive Impariments

**FIGURE 3:**
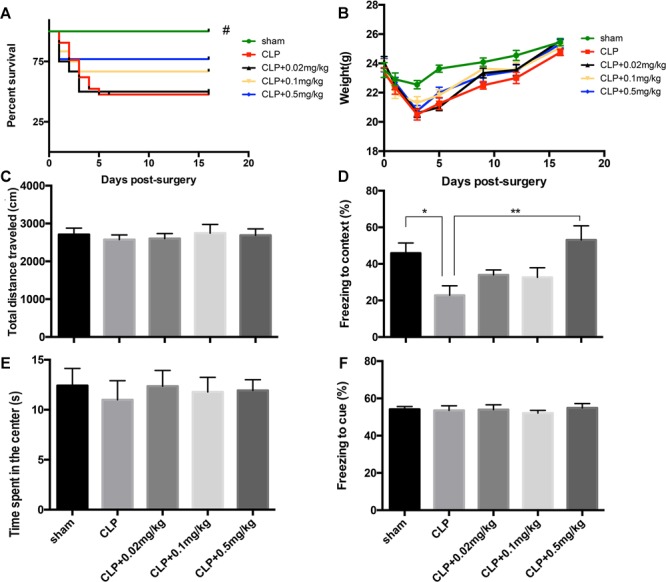
Optimal dose of clenbuterol for the treatment of sepsis-induced cognitive impairments. The survival rate **(A)** was significantly decreased in the CLP group than sham group, while three doses of clenbuterol (0.02, 0.1, and 0.5 mg/kg) treatment did not improve the survival rate of the sepsis. There was no significant difference in weight **(B)**, ambulatory distance **(C),** and time spent in the center **(E)** in the open field test among the five groups at 16 days following CLP. The freezing time to context **(D)** was significantly decreased in the CLP group than sham group, while clenbuterol at 0.5 mg/kg administration significantly increased the freezing time. No significant difference was detected in the cued fear conditioning **(F)** among the five groups. *N* = 9–11 mice/group. Compared with the CLP group, ^#^*p* < 0.05, ^∗^*p* < 0.05, ^∗∗^*p* < 0.01. All data are mean ± SEM.

As revealed in Figure 3, our data showed that 0.5 mg/kg was the optimal dose because it improved contextual memory impairment (*F*_4,44_= 4.463, *p* = 0.004; Figure 3D) without affecting the locomotor activity (total distance: *F*_4,44_= 0.707, *p* = 0.600; time spent in the center of arena: *F*_4,44_= 0.219, *p* = 0.926; Figure 3C,E), the survival rate, and the weight of sepsis mice. For this reason, clenbuterol at the dose of 0.5 mg/kg was used in our subsequent experiments.

### Early Clenbuterol Treatment Attenuated Sepsis-Induced Hippocampus-Dependent Cognitive Impairments

**FIGURE 4:**
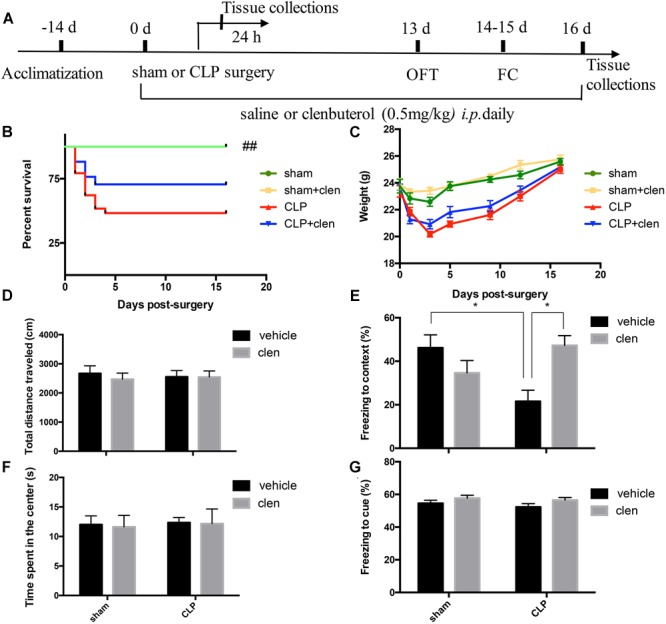
Early clenbuterol treatment attenuated sepsis-induced hippocampus-dependent cognitive impairments. **(A)** Schematic timeline of the experimental procedures. The survival rate **(B)** was significantly decreased in the CLP group than sham group, while early clenbuterol treatment did not improve the survival rate of the sepsis. There was no significant difference at weight **(C)**, ambulatory distance **(D)** and time spent in the center **(F)** in the open field test among the four groups at 16 days following CLP. **(E)** The freezing time to context was significantly decreased in the CLP group than control group, while clenbuterol administration significantly increased the freezing time. No significant difference was detected in the cued fear conditioning **(G)** among the four groups. *N* = 9–11 mice/group. Compared with the CLP group, ^##^*p* < 0.01, ^∗^*p* < 0.05. All data are mean ± SEM.

The open field test was performed at 13 days following operation to determine whether CLP or clenbuterol treatment influences the locomotor activity of mice. There was no difference in interaction between CLP and clenbuterol treatment for the total distance (interaction effect, *F*_1,35_= 0.177, *p* = 0.676; Figure 4D) and time spent in the center of arena (interaction effect, *F*_1,35_= 0.004, *p* = 0.949; Figure 4F) in the open field test. *Post hoc* comparisons revealed that no difference in the total distance (*p* > 0.05) and time spent in the center of arena (*p* > 0.05) was observed among the four groups. As above, early clenbuterol treatment did not improve the survival rate and the weight of the sepsis (Figure 4B,C).

To evaluate sepsis-induced cognitive impairments, the fear conditioning test was performed at 14 days after operation. The interaction between CLP and clenbuterol treatment was significant for contextual freezing time (interaction effect, *F*_1,35_ = 12.04, *p* = 0.001; Figure 4E). *Post hoc* comparisons revealed that the freezing time to context was significantly decreased in the CLP + vehicle group compared with the sham group (*p* < 0.05), which were reversed by clenbuterol treatment (*p* < 0.05). However, there was no difference in interaction between CLP and clenbuterol treatment for the post-tone freezing time in the auditor-cued fear conditioning test (interaction effect, *F*_1,35_= 0.10, *p* = 0.754; Figure 4G). *Post hoc* comparisons revealed that no difference in post-tone freezing time (*p* > 0.05) was observed among the four groups. These data suggested that early clenbuterol treatment reversed hippocampus-dependent cognitive impairments.

### Early Clenbuterol Treatment Promoted Alternative Microglia Activation and Suppressed Neuroinflammation

**FIGURE 5:**
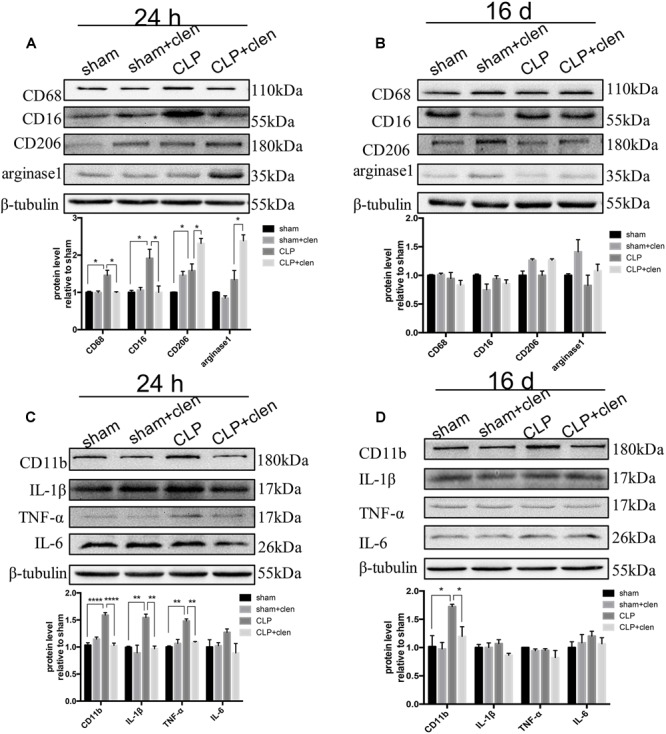
Early clenbuterol treatment promoted alternative microglia activation and suppressed neuroinflammation. **(A)** Clenbuterol drove microglia to an alternative activation state in sepsis mice at 24 h following CLP in the hippocampus. Representative Western blotting bands of CD68 and CD16 and CD206 and arginase1 and quantitative analysis of the relative protein levels. **(C)** Clenbuterol suppressed the proinflammatory response in sepsis mice at 24 h following CLP. Representative Western blots bands of CD11b and inflammatory cytokine IL-1β, TNF-α, and IL-6 and quantitative analysis of the relative protein level. **(B,D)** There was no significant difference in CD68, CD16, CD206, and arginase1 and IL-1β, TNF-α, IL-6 among the four groups at 16 days following CLP. Sepsis increased the expression level of CD11b at 16 days following CLP, which was reversed by early clenbuterol treatmen. *N* = 3 mice/group. Compared with the CLP group, ^∗^*p* < 0.05, ^∗∗^*p* < 0.01, ^∗∗∗∗^*p* < 0.0001. All data are mean ± SEM.

We next investigated whether clenbuterol modulate sepsis-induced inflammatory responses in the hippocampus following CLP. The interaction between CLP and clenbuterol treatment was significant for CD68 (interaction effect, *F*_1,8_ = 9.312, *p* = 0.016; Figure 5A) and CD16 (interaction effect, *F*_1,8_ = 9.888, *p* = 0.014; Figure 5A) at 24 h following CLP. *Post hoc* comparisons showed that the expressions of CD68 and CD16 (M1 microglia markers) were markedly increased in sepsis mice (*p* < 0.05), which were reversed by clenbuterol treatment (CD68: *p* < 0.05; CD16: *p* < 0.05). In addition, the interaction between CLP and clenbuterol treatment was significant for arginase1 (interaction effect, *F*_1,8_= 8.043, *p* = 0.022; Figure 5A), while the interaction between these treatments on CD206 failed to obtain such difference (interaction effect, *F*_1,8_= 1.186, *p* = 0.308; Figure 5A) at 24 h following CLP. *Post hoc* comparisons showed the expressions of CD206 and arginase1 (M2 microglia markers) were dramatically increased in CLP + clenbuterol group compared with CLP + vehicle group (CD206: *p* < 0.05; arginase1: *p* < 0.05). The interaction between CLP and clenbuterol treatment was significant for IL-1β (interaction effect, *F*_1,8_= 8.326, *p* = 0.020; Figure 5C) and TNF-α (interaction effect, *F*_1,8_= 12.63, *p* = 0.008; Figure 5C) at 24 h following CLP. *Post hoc* comparisons showed that the expressions of IL-1β and TNF-α were markedly increased in sepsis mice at 24 h following CLP (all *p* < 0.01), whereas clenbuterol treatment markedly decreased these inflammatory mediators (all *p* < 0.01). There was no significant difference in IL-6 level at 24 h following CLP among the four groups. There was no difference in interaction between CLP and clenbuterol treatment for CD68, CD16, CD206, arginase1, IL-1β, TNF-α, and IL-6 at 16 days following CLP (interaction effect, CD68: *F*_1,8_= 0.914, *p* = 0.367; CD16: *F*_1,8_= 1.538, *p* = 0.250; CD206: *F*_1,8_= 2.149, *p* = 0.181; arginase1: *F*_1,8_= 0.261, *p* = 0.623; IL-1β: *F*_1,8_= 0.289, *p* = 0.606; TNF-α: *F*_1,8_= 0.448, *p* = 0.522; IL-6: *F*_1,8_= 0.1.860, *p* = 0.210; Figure 5B,D). *Post hoc* comparisons revealed that no difference in CD68, CD16, CD206, arginase1, IL-1β, TNF-α, and IL-6 at 16 days following CLP (all *p* > 0.05) was observed among the four groups. Interestingly, the interaction between CLP and clenbuterol treatment was significant for CD11b at 24 h (interaction effect, *F*_1,8_ = 59.21, *p* < 0.001; Figure 5C) and 16 days (interaction effect, *F*_1,8_= 13.92, *p* = 0.006; Figure 5D) following CLP. *Post hoc* comparisons showed that the expression of CD11b was significantly upregulated at 24 h and 16 days following CLP (24 h: *p* < 0.001; 16 days: *p* < 0.05), which could be decreased by clenbuterol treatment (24 h: *p* < 0.001; 16 days: *p* < 0.01).

### Early Clenbuterol Treatment Prevented the Down-Regulated CREB/BDNF Signaling and Reversed the Synaptic Protein Loss

**FIGURE 6:**
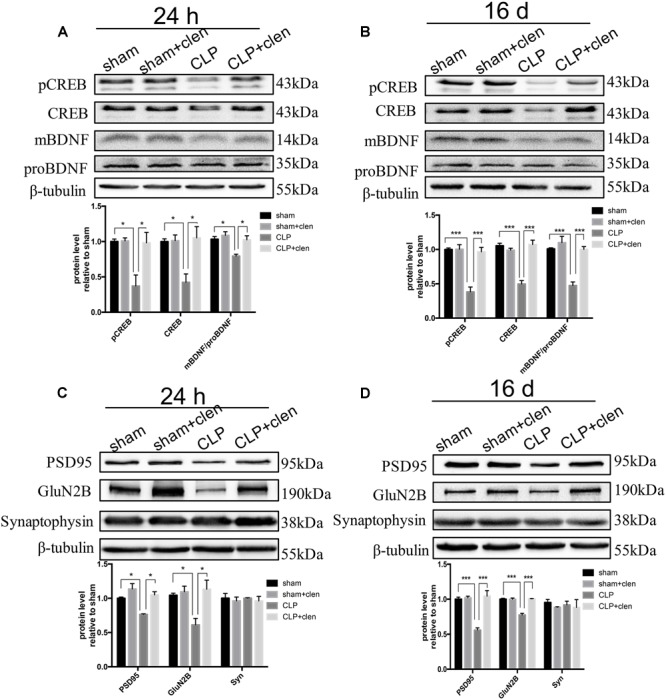
Early clenbuterol treatment prevented the down-regulated CREB/BDNF and reversed the decreased synaptic proteins. **(A,B)** Clenbuterol prevented the down-regulated CREB/BDNF at 24 h and 16 days following CLP in the hippocampus. Representative Western blotting bands of pCREB, CREB, and mBDNF/proBDNF and quantitative analysis of the relative protein levels. **(C,D)** Clenbuterol ameliorated the decreased synaptic protein PSD95 and GluN2B induced by sepsis at 24 h and 16 days following CLP in the hippocampus. Representative Western blotting bands of PSD95, GluN2B, and synaptophysin and quantitative analysis of the relative protein levels. *N* = 3 mice/group. Compared with the CLP group, ^∗^*p* < 0.05, ^∗∗^*p* < 0.01, ^∗∗∗^*p* < 0.001. All data are mean ± SEM.

We next assessed whether clenbuterol upregulated CREB/BDNF signaling pathway in the hippocampus. The interaction between CLP and clenbuterol treatment was significant for pCREB, total CREB (CREB) and the ratio of mBDNF/proBDNF at 24 h following CLP (interaction effect, pCREB: *F*_1,8_= 7.363, *p* = 0.027; CREB: *F*_1,8_= 8.061, *p* = 0.022; the ratio of mBDNF/proBDNF: *F*_1,8_= 6.938, *p* = 0.030; Figure 6A) and 16 days (interaction effect, pCREB: *F*_1,8_= 23.57, *p* = 0.001; CREB: *F*_1,8_= 47.19, *p* < 0.001; the ratio of mBDNF/proBDNF: *F*_1,8_= 14.39, *p* = 0.005; Figure 6B). *Post hoc* comparisons showed that sepsis significantly decreased the expressions of pCREB, CREB, and the ratio of mBDNF/proBDNF at 24 h (all *p* < 0.05) and 16 days (all *p* < 0.001) following CLP, which were significantly reversed by clenbuterol treatment both at 24 h (all *p* < 0.05) and 16 days (all *p* < 0.001) following CLP.

Subsequently, we evaluated the effects of clenbuterol on the synaptic plasticity-related proteins. The interaction between CLP and clenbuterol treatment was significant for GluN2B at 24 h and 16 days following CLP in the hippocampus (interaction effect, 24 h: *F*_1,8_= 7.21, *p* = 0.028; 16 days: *F*_1,8_= 51.86, *p* < 0.001; Figure 6C,D). The interaction between CLP and clenbuterol treatment was also significant for PSD95 at 16 days following CLP (*F*_1,8_= 24.58, *p* = 0.001) but not at 24 h (*F*_1,8_= 2.528, *p* = 0.151). *Post hoc* comparisons showed that sepsis led to significantly decreased expressions of post-synaptic protein PSD95 and GluN2B at 24 h (all *p* < 0.05) and 16 days (all *p* < 0.001) following CLP in the hippocampus, which were significantly attenuated by clenbuterol treatment (all *p* < 0.05) and 16 days (all *p* < 0.001). As indicated in Figure 2B, there was no difference in interaction between CLP and clenbuterol treatment for the presynaptic protein synaptophysin at 24 h (interaction effect, *F*_1,8_= 0.001, *p* = 0.996; Figure 6C) and 16 days (interaction effect, *F*_1,8_= 0.059, *p* = 0.815; Figure 6D) following CLP. *Post hoc* comparisons revealed that no difference in synaptophysin at 24 h and 16 days following CLP (all *p* > 0.05) was observed among the four groups.

### Delayed Clenbuterol Treatment Alleviated Sepsis-Induced Cognition Impairment

**FIGURE 7:**
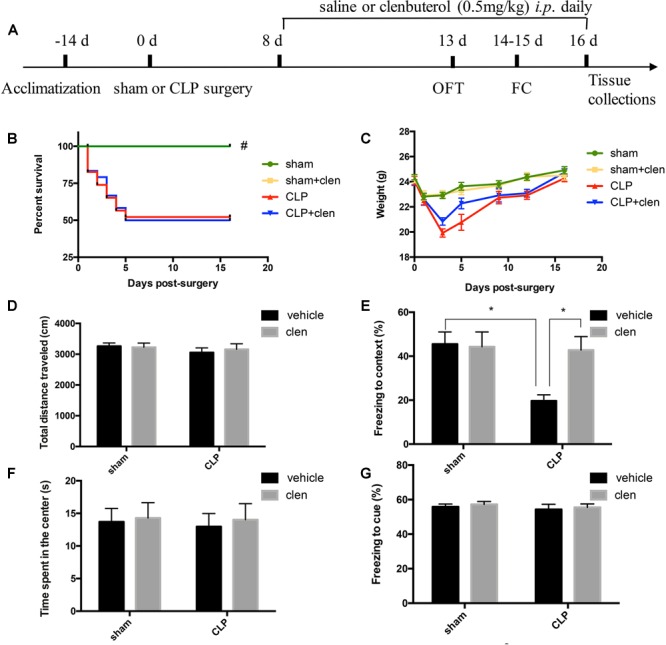
Delayed clenbuterol administration alleviated sepsis-induced cognition impairment. **(A)** Schematic timeline of the experimental procedures. The survival rate **(B)** was significantly decreased in the CLP group than sham group, while delayed clenbuterol administration did not affect the survival rate of the sepsis. There was no significant difference at weight **(C)**, ambulatory distance **(D)**, and time spent in the center **(F)** in the open field test among the four groups at 16 days following CLP. **(E)** The freezing time to context was significantly decreased in the CLP group than control group, while clenbuterol administration significantly increased the freezing time. No significant difference was detected in the cued fear conditioning **(G)** among the four groups. *N* = 9–10 mice/group. Compared with the CLP group, ^#^*p* < 0.05, ^∗^*p* < 0.05. All data are mean ± SEM.

To be more clinically relevant, we determined whether clenbuterol initiated in late sepsis phase could also achieve analogous benefits. As shown in Figure 7, clenbuterol produced similar effects to those observed in sepsis mice with early treatment of clenbuterol. In the fear conditioning test, the interaction between CLP and clenbuterol treatment was significant (interaction effect, *F*_1,35_ = 4.699, *p* = 0.037; Figure 7E). *Post hoc* comparisons revealed that sepsis mice treated with vehicle displayed less freezing time to context than mice in the sham group (*p* < 0.05), which was reversed by clenbuterol treatment (*p* < 0.05). However, there was no difference in interaction between CLP and clenbuterol treatment for the post-tone freezing time in the cued fear conditioning test (interaction effect, *F*_1,35_ = 0.004, *p* = 0.949; Figure 7G). *Post hoc* comparisons revealed that no difference in post-tone freezing time (*p* > 0.05) was observed among the four groups.

### Delayed Clenbuterol Treatment Prevented the Down-Regulated CREB/BDNF Signaling and Reversed the Synaptic Protein Loss

**FIGURE 8:**
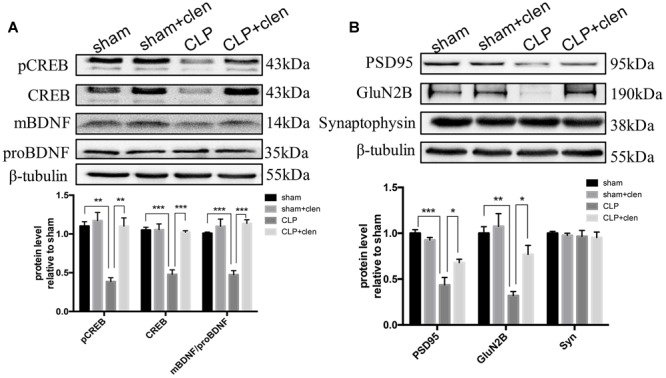
Delayed clenbuterol treatment prevented the down-regulated CREB/BDNF signaling and reversed the decreased synaptic proteins. **(A)** Delayed clenbuterol administration prevented the down-regulated CREB/BDNF signaling. Representative Western blotting bands of pCREB, CREB, and mBDNF/proBDNF at 16 days following CLP in the hippocampus and quantitative analysis of the relative protein levels. **(B)** Clenbuterol ameliorated the decreased synaptic protein PSD95 and GluN2B induced by sepsis. Representative Western blots bands of PSD95, GluN2B and synaptophysin at 16 days following CLP and quantitative analysis of the relative protein levels. *N* = 3 mice/group. Compared with the CLP group, ^∗^*p* < 0.05, ^∗∗^*p* < 0.01, ^∗∗∗^*p* < 0.001. All data are mean ± SEM.

As revealed in Figure 8, the interaction between CLP and clenbuterol treatment was significant for pCREB, CREB and the ratio of mBDNF/proBDNF at 16 days following CLP in the hippocampus (interaction effect, pCREB: *F*_1,8_ = 14.96, *p* = 0.005; CREB: *F*_1,8_ = 27.98, *p* < 0.001; the ratio of mBDNF/proBDNF: *F*_1,8_ = 23.48, *p* = 0.001; Figure 8A). *Post hoc* comparisons revealed that delayed clenbuterol treatment could rescue these downregulated protein expressions (pCREB: *p* < 0.01; CREB: *p* < 0.001; the ratio of mBDNF/proBDNF: *p* < 0.001). In addition, the interaction between CLP and clenbuterol treatment was also significant for PSD95, but not for GluN2B (interaction effect, PSD95: *F*_1,8_ = 9.525, *p* = 0.015; GluN2B: *F*_1,8_ = 3.862, *p* = 0.085; Figure 8B). *Post hoc* comparisons revealed that the decreased expressions of the PSD95 and GluN2B by sepsis were reversed by delayed clenbuterol treatment (all *p* < 0.05). Likewise, there was no difference in interaction between CLP and clenbuterol treatment for the presynaptic protein synaptophysin (interaction effect, *F*_1,8_= 0.003, *p* = 0.960; Figure 8B). *Post hoc* comparisons revealed that no difference in synaptophysin (*p* > 0.05) was observed among the four groups.

## Discussion

In the present study, we showed that the expression of β2-AR was significantly reduced in the hippocampus of sepsis mice. To the best of our knowledge, this was the first demonstration that sepsis induced downregulated expression of β2-AR in the hippocampus. Importantly, we found that early treatment with the brain penetrant β2-AR agonist, clenbuterol, had a neuroprotective effect but was not sufficient to improve survival rate. These data complement previous studies indicating that clenbuterol has neuroprotective actions in rodent models of ischemic stroke (Lechtenberg et al., 2019), Rett syndrome (Mellios et al., 2014). This neuroprotective effect of clenbuterol in the memory impairment induced by sepsis was accompanied by polarizing microglia toward an anti-inflammatory phenotype, reducing proinflammatory cytokines including IL-1β and TNF-α, and reversing synaptic abnormalities. Intriguingly, delayed clenbuterol treatment similarly improved the cognitive impairments by attenuating synaptic abnormalities after sepsis. However, clenbuterol These results support the beneficial effects of both early and delayed clenbuterol treatment.

The noradrenergic transmission system is known to be involved in various forms of learning and memory (O’Dell et al., 2015). Abnormalities of noradrenergic neurotransmission is associated with a number of cognitive disorders including AD (Xu et al., 2018), Down syndrome (Dang et al., 2014), and depression (Manczak et al., 2019). The levels of β2-AR is decreased in AD brain, presumably due to rapid internalization of β2-AR induced by soluble amyloid beta (Aβ) (Li et al., 2013; Takahashi et al., 2015). By contrast, activation of β2-AR signaling has been proved to prevent LTP impairment by soluble Aβ (Wang et al., 2011; Li et al., 2013). These results suggest that β2-AR signaling is required for LTP formation and memory. On the other hand, β2-AR can suppress neuroinflammatory response and induce the neurotrophic factors BDNF and nerve growth factor to protect against kainic acid-induced neurotoxicity (Gleeson et al., 2010). Indeed, there is growing interest in β2-AR as a neuroprotective target for neurodegenerative disease. In our study, we showed that the expression of β2-AR was significantly reduced in the hippocampus after sepsis. Notably, activation of β2-AR can effectively improve sepsis-induced cognitive impairments, which highlights a critical role of β2-AR in sepsis-induced brain dysfunction. However, the mechanism by which β2-AR activation improves sepsis-induced cognitive impariments remains to be determined.

The pathophysiology of cognitive impairments induced by sepsis is likely to be multifactorial. Among these mechanisms, release of proinflammatory mediators within the brain play a vital role (Han et al., 2018). Inflammatory cytokines are crucial mediators of septic and aseptic inflammation (Michels et al., 2019). It has been suggested that acute inflammatory response is responsible for the long-term brain dysfunction observed in sepsis patient (Manczak et al., 2019). This finding is further confirmed by previous studies that IL-1 impairs context-associated fear memory (Goshen et al., 2007) and IL-1 receptor antagonist is protective against the hippocampus-dependent cognitive deficits induced by systemic lipopolysaccharide (LPS) administration (Terrando et al., 2010). Apparently increased levels of IL-1β was observed in the hippocampus of septic mice at 24 h following CLP, which remained high by 9 days and returned to control levels by 30 days (Moraes et al., 2014). Our previous study showed sepsis-induced increase in IL-1β level in the hippocampus peaked at 7 days after CLP (Ji et al., 2018). Also our current study showed that significant high levels of IL-1β in the hippocampus of septic mice at 12 h, remaining elevated at 7 days following CLP. Our results together with previous findings suggest that neuroinflammation plays a key role in sepsis-induced cognitive impairments.

As resident macrophages of the CNS, microglia are well-known as a sensor of tissue injury or infection and are pivotal in the pathogenesis of neurodegenerative diseases (Fu et al., 2016; Cao et al., 2018). Activated microglia have dual functions, the classical (M1) phenotype and the alternative (M2) phenotype. M1 activation induces the release of proinflammatory cytokines, hindering CNS repair and expanding tissue damage, while M2 activation produces antiinflammatory mediators involved in brain recovery (Bacou et al., 2017; Wang et al., 2017). Previous studies demonstrated that stimulation of β2-AR promoted an antiinflammatory phenotype in glial cells (Gleeson et al., 2010). In addition, β2-AR signaling has been demonstrated to be able to regulate macrophage activity in cancer via mechanisms that could be interpreted as M2-promoting and/or M1-suppressing effects (Trowsdale, 2002). An *in vitro* study showed that β2-AR activation dampened the LPS-induced M1 polarization in macrophages (Bacou et al., 2017). Another study confirmed that the peripheral effects of clenbuterol can be dissociated from its central effects and the increased expression of the antiinflammatory molecules IL-1ra and IL-1RII may serve to limit the effects of clenbuterol-induced IL-1β on brain function and behavior (Ryan et al., 2016). In the present study, we extended these prior studies *in vivo* by demonstrating that activation of the β2-adrenergic receptor not only regulated the production of inflammatory cytokines but also modified the entire microglia phenotype, resulting in an M2 regulatory microglia phenotype. This is reflected by the observations that M1-associated markers (CD68 and CD16) were significantly upregulated while M2-associated markers downregulated (CD206 and arginase1) at 24 h after sepsis, which were reversed by clenbuterol treatment. These data raise the possibilty that clenbuterol treatment suppressed inflammatory mediators IL-1β and TNF-α by promoting alternative microglia activation and exerting neuroprotective effects in sepsis-induced cognitive impairments, although we cannot exclude its possible peripheral anti-inflammatory effects.

Clenbuterol-mediated activation of β2-adrenergic receptors in the brain is well-known to activate PKA, thereby phosphorylating and activating CREB, which subsequently facilitates the transcription of key proteins required for activity-dependent plasticity (Koppel et al., 2018), especially for BDNF (Zhen et al., 2019). It is well-established that activation of the CREB/BDNF signaling is crucial for memory enhancement (Karthivashan et al., 2018). It is reported high level of inflammatory cytokines can negatively regulate BDNF level and impair hippocampal-dependent memory (Tapia-Arancibia et al., 2008), whereas blockade of inflammation can restore some of these alterations (Han Y. et al., 2019). Moreover, it has been shown that IL-1β impaired pCREB and BDNF, which are crucial for synaptic plasticity (Tong et al., 2012). Besides IL-1β, TNF-α has also been demonstrated to downregulate the pCREB and BDNF (Arai et al., 2005; Xu et al., 2015). In our study, we found the expressions of pCREB, total CREB and the ratio of mBDNF/proBDNF began to decrease at 24 h following CLP and continued to be lower until the end of the study. Synaptic proteins such as PSD95 and GluN2B have key function in learning and memory and their disruptions correlate well with cognitive impairments (Zhang et al., 2017; Merino-Serrais et al., 2019). In one previous study, significantly decreased levels of synaptophysin and PSD95 were observed in the hippocampus of sepsis mice at 24 h and 3 days following CLP (Moraes et al., 2014), while another study indicated that sepsis selectively decreased the levels of GluN2B but not synaptophysin at the first week after CLP or LPS (Zhang et al., 2017). Consistently, we showed that CLP had a long-term deleterious impact on the hippocampal post-synaptic protein PSD95 and GluN2B but did not affect the presynaptic protein synaptophysin. However, another study found a single *i.p.* injection of LPS induced synaptophysin loss lasted at least for 2 months after sepsis (Weberpals et al., 2009). The discrepancy could be explained by different animal models used and time of protein determination. Notably, our study showed that both early and delayed clenbuterol treatment improved the cognitive impairments by reversing synaptic abnormalities after sepsis. Although the causal relationship was not established, it is possible that sepsis induced enhanced hippocampal inflammatory response, which subsequently downregulated CREB/BDNF signaling, synaptic protein loss and ultimately resulted in hippocampus-dependent cognitive impariments.

There are a few limitations in our study. It should be mentioned that we only studied the hippocampus, which cannot represent the whole brain change of inflammation and synaptic plasticity. However, we selected this brain region because the hippocampus plays a key role in learning and memory (Hagena et al., 2016) and is more susceptible to sepsis (Semmler et al., 2005; Annane, 2009). In addition, it has been suggested that β2-AR is expressed predominantly in glia (Catus et al., 2011). Therefore, further research is necessary to determine the specific distribution of β2-AR. Moreover, clenbuterol, a long-acting brain penetrant β2-AR agonist, was applied in the present study. Nevertheless, using mice overexpressing β2-AR in the hippocampus will provide more compelling evidence with regard to whether β2-AR can influence the neuroinflammatory response and synaptic plasticity after sepsis.

In summary, sepsis can elicit neuroinflammation, synaptic protein loss and cognitive impairments, which is partly attributed to the dysfunction of β2-AR signaling in the hippocampus. Pending further studies, activation of β2-AR represents a promising neuroprotective approach to modulate microglial phenotype, restore synaptic plasticity and treat sepsis-induced hippocampus-dependent cognitive impairments.

## Data Availability

The raw data supporting the conclusions of this manuscript will be made available by the authors, without undue reservation, to any qualified researcher.

## Ethics Statement

All procedures were carried out based on the Guideline for the Care and Use of Laboratory Animals from the National Institutes of Health (Bethesda, MD, United States). Experiments were performed in accordance with protocols approved by the Ethics Committee of Jinling Hospital, Nanjing University, China.

## Author Contributions

M-MZ carried out the behavioral study and drafted the manuscript. M-HJ, MJ, and HT performed the immunohistochemistry and Western blotting analysis. Z-QZ and M-HJ participated in the design of the study and statistical analysis. J-JY guided the study design and helped to draft the manuscript. All authors read and approved the final manuscript.

## Conflict of Interest Statement

The authors declare that the research was conducted in the absence of any commercial or financial relationships that could be construed as a potential conflict of interest.
